# Antibacterial Effect of Curcumin against Clinically Isolated *Porphyromonas gingivalis* and Connective Tissue Reactions to Curcumin Gel in the Subcutaneous Tissue of Rats

**DOI:** 10.1155/2019/6810936

**Published:** 2019-09-30

**Authors:** Aram Mohammed Sha, Balkees Taha Garib

**Affiliations:** ^1^Department of Periodontology, College of Dentistry, University of Sulaimani, Sulaimani, Kurdistan Region, Iraq; ^2^Department of Oral Pathology, College of Dentistry, University of Sulaimani, Sulaimani, Kurdistan Region, Iraq

## Abstract

The aim of this study was to find the antibacterial potential of curcumin against *Porphyromonas gingivalis* and connective tissue responses to curcumin gel in the subcutaneous tissue of rats. The sample consisted of subgingival plaque collected from patients with chronic periodontitis. The *P. gingivalis* clinically isolated strain was confirmed by anaerobic culture, morphology, biochemical tests (Vitek ANC Kit), and PCR (16S rDNA). Minimum inhibitory concentration (MIC) and minimum bactericidal concentration (MBC) were determined by incubation of twofold serial dilution of broth media containing curcumin (from 100 to 0.05 *µ*g/ml) for 48 h at 37°C. Fifteen adult Wistar rats (3-4 months old) were used and randomly divided into three groups (negative control, positive control, and experimental groups). Tubes were implanted on the back skin (45 tubes). Rats were euthanized at 7, 30, and 60 days after surgical processes, and then the samples were taken and processed to achieve conventional hematoxylin and eosin-stained slides. The MIC and MBC of curcumin against clinically isolated *P. gingivalis* were 12 *µ*g/ml. Curcumin gel caused moderate inflammatory reactions at 7 and 30 days, while at 60 days, it caused dramatic decline and resulted in a nonsignificant response. Besides, curcumin gel stimulated quick reepithelialization, fibroblast proliferation, and scarring through the formation of thick bundles of well-organized collagen fibers. Curcumin has an effective antibacterial action against clinically isolated *P. gingivalis* at low concentration (12 *µ*g/ml), and it was regarded as the biocompatible material in the subcutaneous tissues.

## 1. Introduction

Curcumin (diferuloylmethane) is an orange-yellow pigment obtained from the rhizomes of *Curcuma longa*. Chemically, it is an extended pseudosymmetric polyphenol (diferuloylmethane) [1]. In recent years, in vitro and in vivo researchers have proposed that curcumin has anticarcinogenic, antioxidant, and anti-inflammatory effects [2, 3] besides antibacterial, antiviral, and antifungal properties [4] and besides being a wound-healing accelerator [5].

The strict anaerobic, rod-shaped Gram-negative bacterium *P. gingivalis* is one of the key pathogens causing chronic periodontitis [6] and lately has been known as a keystone pathogen [7]. Furthermore, it has been found that *P. gingivalis* exerts an impact on systemic health, e.g., cardiovascular diseases and rheumatoid arthritis [8, 9]. *P. gingivalis* can penetrate gingival tissues and cannot be removed by mechanical debridement [10]. Thus, it provides the chance for recolonization of periodontal pockets and recurrence of the disease. An adjunct treatment of local drug delivery has been suggested for such periopathogens [11].

Many antimicrobial agents have been used successfully as an adjunct for the elimination of such periopathogens including tetracycline, chlorhexidine, and metronidazole [12, 13, 14]. However, the success was not permanent because of the development of multidrug-resistant microorganisms, interbacterial transfer of resistance determinants, and various side effects [15]. The use of medicinal plants in periodontics as natural antimicrobial products attracted the attention of researchers as they offer a viable alternative adjunct therapy to mechanical plaque removal. Curcumin is one of the natural products that has been examined for that purpose.

Both in vitro and in vivo tests can be used to evaluate the biological effects of new materials. The in vitro tests assess the characteristics and reactions of the materials directly in the cell culture, and the response is then evaluated. The in vivo tests depend on the subcutaneous, bone, or intramuscular implantation of the material in animals. Within specific time intervals, the material-tissue interface is examined macroscopically and microscopically for any pathological or physiological alterations. The early event after tested material implantation is an inflammatory response, which is the main characteristic finding [16].

On the contrary, a wound-healing process is multifarious and includes the coordination of several processes. Microbial infections and uncontrolled inflammatory reactions (due to continuous microbial infections) can postpone wound healing [17]. Therefore, any product that could control both microbial proliferation and inflammatory reactions would be useful for quickening wound healing [18]. Curcumin is one of the few natural products that possess a broad range of pharmacological characteristics with high curative effects.

Bacterial growth states from in vivo ecosystems, including bacterial infections, are conspicuously different from those obtained under in vitro conditions. The multispecies residential areas in complex and altering environments of the actual world contrast with the standardized and idealized conditions in laboratory nomenclature. However, in spite of these differences, microbial research is mostly dependent upon laboratory strains that are fundamentally nonpathogenic [19].

Numerous studies have shown the antibacterial effect of curcumin against laboratory strains of *P. gingivalis* [20–23] (ATCC 33277), but a little information regarding the antibacterial effect of curcumin against the clinically isolated strain was available and curcumin gel should be able to be biocompatible with the surrounding tissues, when it is applied locally by the same dose of antibacterial effect. Therefore, the aim of this study is to find the antibacterial effect of curcumin against the clinically isolated strain of *P. gingivalis* and investigate connective tissue responses to curcumin gel in comparison with the empty tube (negative control) and tetracycline tube (positive control).

## 2. Materials and Methods

### 2.1. Plaque Sampling

The study was performed after getting approval from the Ethics Committee for Human Research of Sulaimani University, Iraq. Five healthy patients who had generalized chronic periodontitis with periodontal pockets of 6 mm depth or more, untreated for at least six months, were selected for collection of subgingival plaque samples.

The selected pocket was isolated by cotton rolls. Any supragingival plaque and calculus were removed by a sterile periodontal curette. Then, a sterile paper point (F2 Dia-ProT™) was inserted slowly into the periodontal pocket until tissue resistance was felt and left in place for 60 sec. After that, they were carefully removed and immediately streaked over supplemented Columbia agar plates. Inoculated plates were placed in an anaerobic condition created by AnaeroPack®-Anaero (MGC, Japan) and an anaerobic jar (BBL® GasPak system) and incubated for 7–10 days at 37°C. Patients' consents and approvals were obtained prior to collecting the samples.

### 2.2. Composition of Media

The composition of media per 500 ml was as follows: Columbia agar base (21.25%) (code LAB001, UK), 5 *µ*g/mL of hemin (1.5 mg) (Sigma-Aldrich, China), 1 *µ*g/mL vitamin K1 (0.5 mg) (Himedia), 5% human blood (25 ml), colistin methanesulfonate (7.68 mg), bacitracin (5 mg) (Himedia), nalidixic acid (7.5 mg) (Himedia), and distilled water [24].

Media were prepared and stored under strictly anaerobic conditions to prevent oxidation.

### 2.3. Identification and Isolation of Microorganisms

The *P. gingivalis* identification was based on colony morphology, pigment production, Gram staining, Vitek 2 system (ANC Kit, bioMerieux), and aerobic control and finally confirmed by the PCR technique as described below, and the proved isolated strain was stored at −80°C.

 DNA isolation: a colony was taken from each sample and mixed with 50 *µ*L of sterilized ultrapure deionized distilled water (ddH_2_O) in an Eppendorf test tube, vortexed well until homogenized, and then incubated in a heat block for 10 min at 95°C. After that, the samples were spun down, and the supernatant (DNA) was used as a template.
 DNA amplification was performed using specific primer pairs targeted at the 16S rDNA gene to confirm the presence of *P. gingivalis* [25]:
   5′-AGGCAGCTTGCCATACTGCG-3′
   5′-ACTGTTAGCAACTACCGATGT-3′
 PCR preparation was performed in a final volume of 20 *μ*l (containing 2 *μ*l of the reverse primer (10 pmol/*μ*l), 2 *μ*l of the forward primer (10 pmol/*μ*l), 10 *μ*l of 2X Prime Taq Premix (GeNet BioG 2000), 1 *μ*l of ddH_2_O, and 5 *μ*l of the DNA template). The DNA sample initially was denaturated for 5 min at 95°C for one cycle followed by an amplification step which was repeated for 35 cycles (denaturation of the DNA template at 95°C for 30 sec, annealing of the specific primers at 62°C for 30 sec, and extension of primers at 72°C for 30 sec). The final extension was done for 5 min at 72°C for one cycle in a PCR thermocycler (Veriti™ 96-well thermal cycler, Applied Biosystems, USA).

The PCR product was analyzed by 2% agarose gel electrophoresis at 80 V for 35 min. The gel was stained with 3 *μ*l of ethidium bromide. A 100 bp plus DNA marker (cat. no. M 2000) was used as a molecular weight marker. Gel purification was performed for the bands by using the GeneJET™ Gel Extraction Kit (#K0691) from Fermentas, UK. In addition, the standard sequencing for the PCR product was done (Macrogen, South Korea).

### 2.4. Antibacterial Effect Assay

All assays were performed in triplicate.

### 2.5. Determination of MIC

The 95% curcumin (BulkSupplements Pure Curcumin 95% Natural Turmeric Extract Powder) was serially diluted, in a range from 100 to 0.05 *μ*g/ml in twelve test tubes with bacterial suspension (5 × 10^5^ CFU/ml) by using 0.5 McFarland's turbidity standard. Tetracycline (6 *μ*g/ml) was used as a positive growth control, and a broth solution was used as a negative growth control. The stock solution of the curcumin was dissolved in dimethyl sulfoxide to obtain 200 *μ*g/ml.

**Figure 1:**
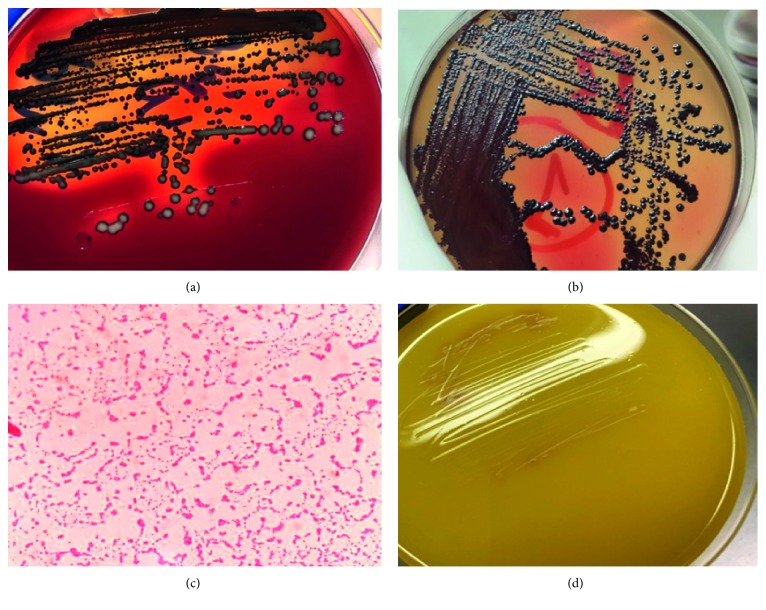
Pure isolated bacterial colonies of *P. gingivalis* after 7 (a) and 10 (b) days on Columbia agar appearing as round, smooth, shiny, and convex black colonies. (c) Gram-negative short rods of *P. gingivalis* and (d) agar formed media cultured from the tube with the MIC of curcumin showing no *P. gingivalis* growth.

For MIC, twelve dilutions of the curcumin were prepared with Mueller Hinton broth (LAB114) (1 ml broth/tube) using the twofold serial dilution method: 100, 50, 25, 12.5, 6.25, 3.12, 1.6, 0.8, 0.4, 0.2, 0.1, and 0.05 *μ*g/ml. To each of the 12 prepared MIC tubes, varying concentrations of curcumin were incorporated. Then, the tubes were inoculated with a 100 *μ*l strain of clinically isolated *P. gingivalis* so that the final volume was 1100 *μ*l/tube. The tubes were sealed with cotton and incubated for ≥48 h at 37°C in an anaerobic jar using AnaeroPack-Anaero and checked for turbidity (bacterial growth). The minimum concentration of the curcumin in the tube with no turbidity was considered the MIC. The MIC assay was performed in triplicate and finally confirmed by the absence of *P. gingivalis* growth after culturing on agar formed media (Figure 1(d)).

Lastly, the MBC was determined by selecting the concentrations that showed no bacterial growth during the evaluation of the MIC. A sample was taken from the content of the chosen tubes using a bacteriological wire loop, subcultured on Columbia agar plates, and finally incubated in an anaerobic jar for ≥48 h at 37°C. Then, the presence or absence of bacterial growth was determined.

### 2.6. Experimental Animal Design

Fifteen male Wistar albino rats aged 3-4 months and weighing 250 ± 30 g were selected for this study. The study was performed according to the institutional guidelines in accordance with the Ethics Committee for Animal Research of Sulaimani University, Iraq. The animals were housed in temperature-controlled rooms, inside plastic cages identified by their group types and periods. The cages were cleaned daily. The animals have been fed on an ad libitum basis.

After anesthetizing the animals (using the IM injection of a mixture of ketamine hydrochloride 3.3 ml and xylazine hydrochloride 2 ml, in a dose of 0.05 ml/kg of body weight), their backs were shaved and disinfected (5% iodine solution). Three separate incisions (2 cm apart) on the back of each animal were made (No. 15 sterile blade) in a head-tail alignment. Lateral to the incisions, the cutaneous tissue was dissected, and three open-ended polyethylene tubes (1.5 mm internal diameter and 10 mm length) were implanted. Two of them were filled with previous preparations: the first tube was filled with curcumin gel (12.5 *μ*g/ml) (experimental group), the second one was filled with tetracycline gel (6 *μ*g/ml) (positive control group), while the third tube was kept empty (negative control group). The wound was sutured by 4-0 Silk, and tissue response was studied in 3 periods.

### 2.7. Histological Assessment

In each examination period (7, 30, and 60 days after surgical implantation), five animals were euthanized by administrating high doses of anesthetics. The implantation area was reshaved and the skin was disinfected as described previously. The skin and underlying connective tissue containing the implanted tube were excised and fixed in 10% formalin for at least 48 hrs. After fixation, the tissue samples were processed routinely for paraffin-embedded blocks, and 5 *μ*m sections including the long axis of the tube were obtained and stained with hematoxylin and eosin (H&E).

**Figure 2:**
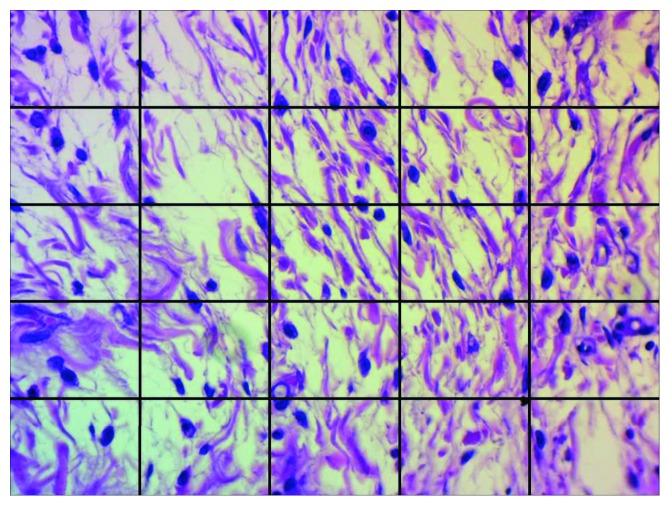
A digital photograph of a subcutaneous tissue reaction at a tube opening divided by a grid into 25 squares for inflammatory cell counting (H&E, ×400).

Quantitative assessment, for tissue response at the opening ends of the implanted tubes, was performed at 400-fold magnification using a light microscope equipped with an image analyzing system (Motic microscope with ToupView (×86, v3.7.4183, 2014; ToupTek)). Each captured image was divided by a grid containing 25 squares (Figure 2). Inflammatory responses were identified and counted by two observers blindly twice, and the average value was calculated. The severity of the inflammatory reaction was scored as follows: 0, <25 cell count (no reaction); +1, >25 and <50 cell count (mild reaction); +2, >50 and<100 cell count (moderate reaction); and +3, >100 cell count (severe reaction).

### 2.8. Statistical Analysis

The inflammatory cell count was presented as mean ± standard deviation and frequency distribution of scores. One-way ANOVA followed by the Tukey test was carried out to ascertain the significance of differences for a parametric variable using the statistical software (PASW Statistics for Windows v21; SPSS Inc.). The level of significance was fixed at a *p* value less than or equal to 0.05.

## 3. Results

The present study showed that 60% (*n* = 3) of the studied samples harbored black-pigmented anaerobic bacteria. Morphologically, after 48 hours, the colonies were small, round, and opaque and showed convex growth. The black-pigmented colonies developed after 7–10 days on lysed blood (Figures 1(a) and 1(b)). The identification of different organisms by application of the automated microbiology system (Vitek 2 system) provided 97% probability for the existence of *P. gingivalis*. Their morphological and biochemical identification features are summarized in Table 1.

**Figure 3:**
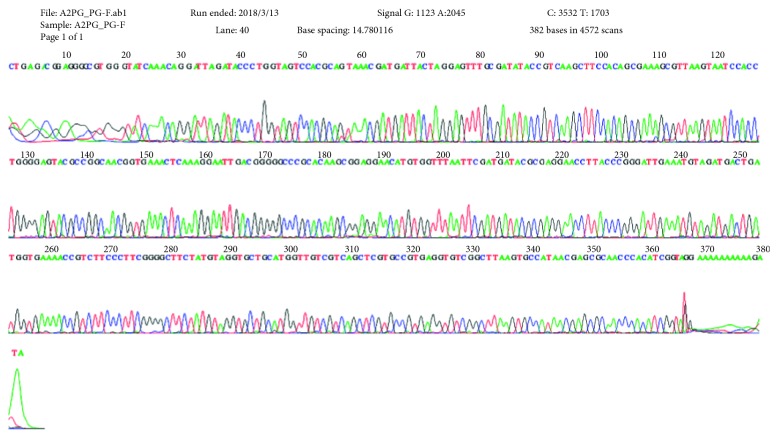
16S rDNA gene sequence of the purified colonies of *P. gingivalis* done in Macrogen, South Korea.

The result of molecular identification using agarose gel electrophoresis showed that all of the three bacterial isolates had a band localized at 404 bp, similar to the template size of *P. gingivalis.* Furthermore, the result of the DNA sequencing demonstrated that 100% of the purified colonies were genetically identical to the ATCC 33277 strain (Figure 3 and the Appendix section).

The MIC and MBC values of curcumin against clinically isolated *P. gingivalis* were both 12.5 *μ*g/ml.

### 3.1. Histopathological Evaluations of Cutaneous Incised Wound Healing

#### 3.1.1. Histopathological Evaluations on the 7^th^ Day

**Figure 4:**
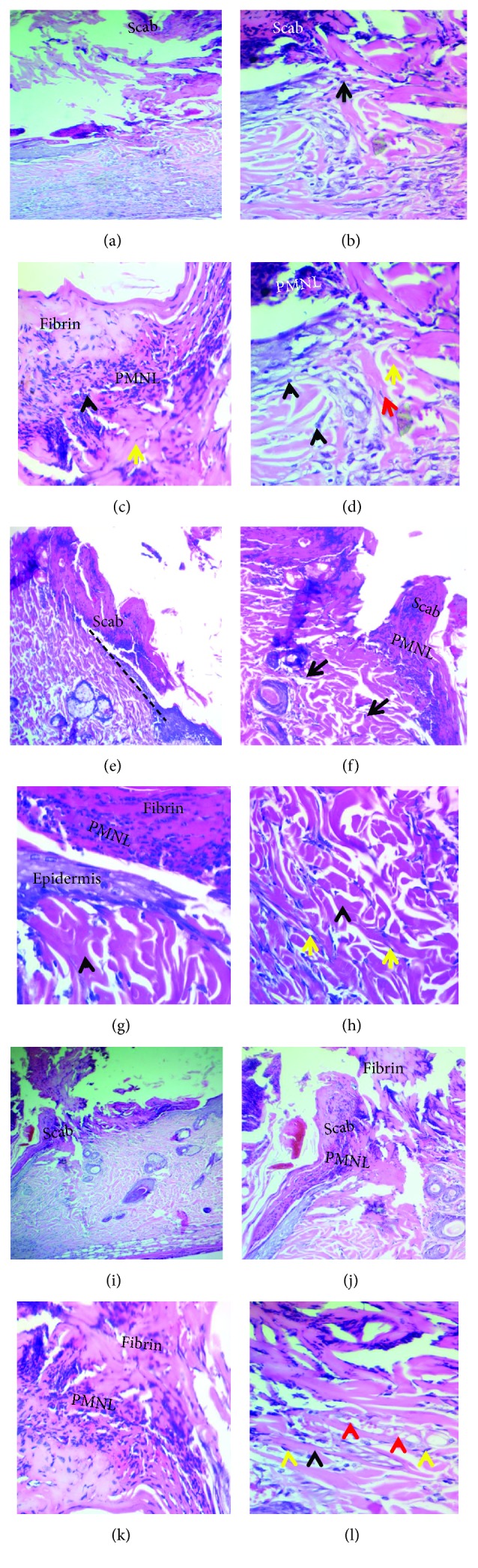
Cutaneous wound healing at seven days. (a–d) Negative control group showing incompletely closed wound covered by the scab and necrotic debris with marked PMNL infiltration (black arrowheads), loss of the matrix, the presence of disorganized collagen fibers (yellow arrows), and proliferating fibroblasts (red arrow). (e–h) Tetracycline group showing a clear line of demarcation (black dashed line), composed of fibrin and PMNLs bridging the whole incision. There is PMNL infiltration in the dermis near the incised wound with the proliferating fibroblasts (yellow arrows) and newly formed unorganized collagen formation (black arrowheads). (i–l) Curcumin group showing the wound completely closed by the scab with the presence of the necrotic debris on the surface and mild-moderate degree PMNL infiltration forming a band between the dermis and the wound, with excessive amount of newly formed collagen fibers (black arrowhead), new capillaries (red arrowheads), and proliferating fibroblasts (yellow arrowheads) (H&E stain, x100 and x200).

Histological examination of tissue sections for wound healing on the 7^th^ day showed a variable degree of epidermal and connective tissue responses corresponding to the implanted material. The cutaneous wound in the empty tube (negative control group) did not oppose completely and showed a mixture of debris, necrotic cells, and fibrin depositions (scab), attached with the inflammatory reactions in the dermis and hypodermis. Besides, new, unorganized collagen fibrils with active fibroblasts were seen within the dermis (Figures 4(a)–4(d)). In the tetracycline group, a demarcation (separating) line between the scab and the dermis was observed, and the wound was considered closed when compared with the previous group. There was a mild infiltration of mixed inflammatory cells in the epidermis extended to the dermis associated with newly formed unorganized collagen fibrils and active fibroblasts (Figures 4(e)–4(h)).

While the wound-healing progression in the curcumin group showed completely opposed wound by the fibrin network that filled out the incisional wound, this network produced a scaffold for wandering active fibroblasts and formed the hyperplastic epidermis. The wound incision in the dermis and epidermis was rich in mixed inflammatory cells; thus, a demarcation line was seen separating the superficial necrotic tissue from underlining vital tissue, containing new blood vessels. Furthermore, the dermis showed more prominent active fibroblasts with an excessive amount of newly formed collagen fibrils (Figures 4(i)–4(l)).

Considering the severity of inflammation at the tube opening, the mean values of the total inflammatory cell count in the subcutaneous tissue and severity score distributions are presented in Tables 2 and 3, respectively. The grade of severity but not the mean score of inflammation severity showed a significant difference (*p* ≤ 0.001) among the three groups. Thus, the control group's scoring revealed moderate inflammatory reaction of 80%, while in the tetracycline group, it was 60% in comparison with the curcumin group that revealed 40% severe inflammatory reaction (Table 2).

On the contrary, the mean of the total cell count was significantly higher in the curcumin group (99 ± 11.82) than in the control (64 ± 20.24) (*p*=0.012) and tetracycline (55 ± 14.44) (*p*=0.002) groups (Table 3).

#### 3.1.2. Histopathological Evaluations on the 30^th^ Day

**Figure 5:**
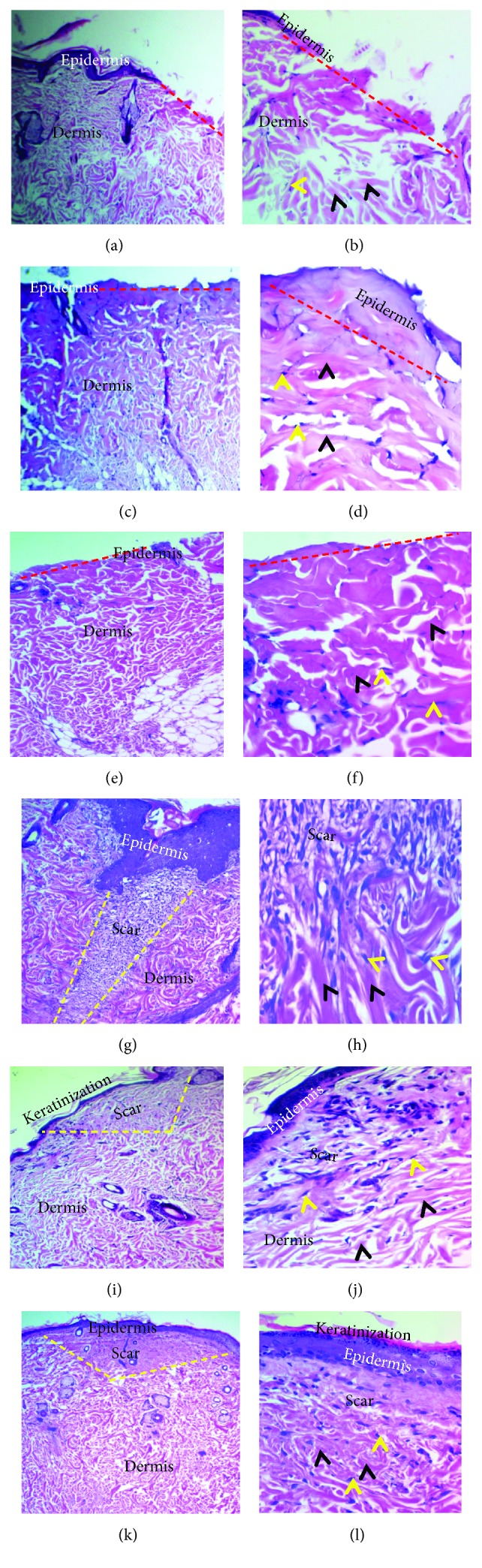
Light micrograph tissue sections of cutaneous wound-healing processes at 30 (a–f) and 60 (g–l) days. (a, b) In the control group, early reepithelialization was detected by forming the immature disorganized epidermis and completely closing the gap (red dashed lines), which resulted in the increase in the thickness of the dermal layer by fibers (black arrowheads) with active fibroblasts (yellow arrowhead) (H&E stain, ×40 and ×200). (c, d) Tetracycline group displayed the late stage of proliferative phase with the typical hyperplastic epidermis (red dashed lines), with increasing thickness of the dermal layer by intense, well-arranged mature bundles of collagen fibers (black arrowheads) with active fibroblasts (yellow arrowheads) (H&E stain, ×40 and ×200). (e, f) Curcumin group presented the late stage of proliferative phase containing the hyperplastic epidermis (red dashed lines) with well-organized collagen fibers in the dermal layer, with increase in thickness of the dermal layer by extreme, mature bundles of collagen fibers (black arrowheads) with active fibroblasts (yellow arrowheads) (H&E stain, ×40 and ×200). (g, h) Control group had uneven minimum keratinized epidermis. The dermis showed moderate-marked density scar tissue (yellow dashes lines) in the center of the wound, infiltration of inflammatory cells, and well-organized thick bundles of collagen fibers (black arrowheads) with fibroblasts (yellow arrowheads) (H&E stain, ×100 and ×200). (i, j) Tetracycline group showed the well-organized mild keratinization epidermis that displayed rete ridges. The dermis consisted of mild-moderate scar (yellow dashed line), minimum inflammatory cell infiltration, dermal papillae, and well-organized thick bundles of collagen fibers (black arrowheads) with fibroblasts (yellow arrowheads) (H&E stain, ×40 and ×200). (k, l) In the curcumin group, there was well-organized keratinized epidermis with rete ridges, mild-moderate scar severity (yellow dashed line), well-organized thick bundles of collagen fibers (black arrowheads), and fibroblasts (yellow arrowheads) (H&E stain, ×40 and ×200).

Histological examination of tissue sections for wound healing on the 30^th^ day showed visible reepithelialization. However, in the control group, an early phase of the reepithelialization process was detected. The immature, discontinued/hyperplastic, and disorganized epidermis overlying the wound's area was associated with an increase in dermal layer thickness by deposition of unorganized collagen fibers produced by active fibroblasts (Figures 5(a) and 5(b)). In the tetracycline group, reepithelialization was completed (late phase), and with the typical hyperplastic epidermis, collagen fibers were thicker and denser (Figures 5(c) and 5(d)), while in the curcumin group, the healing became more pronounced and the epidermis was the mature, hyperplastic, and organized epidermis overlying the area of the wound with an increase in the thickness of the dermal layer by the presence of mature collagen fibers as the bundles with active fibroblasts (Figures 5(e) and 5(f)).

At this period, a significant difference in the mean score of inflammation (*p*=0.016) was seen among groups, being highest in the curcumin group (1.8) and lowest in the tetracycline group (0.8) (*p*=0.017). Again, the grade of inflammation severity significantly differed (*p* ≤ 0.001). Both control and tetracycline groups showed shifting toward no inflammatory response (20%), while the curcumin group did not show a severe reaction, but it showed the highest moderate inflammatory reaction (80%).

The numbers of inflammatory cells significantly decreased (*p*=0.01 or *p* ≤ 0.001) in all groups on the 30^th^ day in comparison with the results of 7 days. The total number of inflammatory cells significantly differed among the groups. It ranged from 32 ± 9.42 in the tetracycline group to 70.4 ± 18.2 in the curcumin group (Table 3).

#### 3.1.3. Histopathological Evaluations on the 60^th^ Day

The control group showed an early remodeling phase. The wounds were fully reepithelialized with early evidence of surface keratinization. Besides, a dense scar tissue was mildly infiltrated by inflammatory cells (neutrophils, macrophages, and lymphocytes), and well-organized thick collagen bundles were observed (Figures 5(g) and 5(h)). In contrast, in the other two groups, the wound showed a late stage of remodeling phase with full maturation of the wound and final steps of dermal reorganization at variable levels. Thus, they have sophisticated wound features such as the well-organized epidermis with dermal papillae, reduced scar tissue (mild-moderate) with a minimum degree of inflammatory cell infiltration, and well-organized thick bundles of collagen fibers (Figures 5(i) and 5(j) for the tetracycline group and Figures 5(k) and 5(l) for the curcumin group).

At the end of the experiment (60 days), the means of the score of inflammation were reduced in all groups with no statistical differences among them. Yet the grading of severity distribution showed significant differences, with the curcumin group being devoid of inflammation (100%), while the control and tetracycline groups revealing 40% and 20% mild reactions, respectively (Table 2).

The mean number of inflammatory cells diminished in the curcumin group (15.3 ± 1.98), and it was nonsignificantly lesser than that in control (24.2 ± 8.75) and tetracycline (22.5 ± 7.32) groups (*p*=0.335).

It is interesting to mention that the reduction in the inflammatory score in the control group or curcumin group was not significant between 7 and 30 days, but it was markedly decreased on the 60^th^ day. On the contrary, the tetracycline group showed noticeable reduction from the 7^th^ to the 30^th^ day but no more significant reduction on the 60^th^ day (Table 4).

Differences in the total cell count depend on experimental periods, indicating that reduction in the control group did not reach a significant level, whereas the tetracycline group had only a marked reduction in the 30-day period. Curcumin showed a significant reduction in inflammatory cell counts among the three studied periods (Table 4).

## 4. Discussion

*P. gingivalis* can produce a high number of virulence factors. It is supposed to be the core pathogen and essential microbiological indicator in the initiation and progression of periodontal disease [26], and as it was mentioned earlier, *P. gingivalis* was considered a keystone pathogen of periodontal diseases [7]. Therefore, a new study was directed toward the antibacterial effect of various natural products against this pathogen. For example, a study by Azeez and Gaphor [27] reported the antibacterial effect of essential oil extracted from the gum of *Pistacia atlantica Kurdica* against *P. gingivalis*.

Recently, a new study by Singh et al. reported the antibacterial effect of curcumin against clinically isolated *P. gingivalis* through inhibition of gingipains R and K which are necessary for proliferation of *P. gingivalis* and aggravation of lesions in chronic periodontitis [28]. However, the biocompatibility of curcumin was conducted in vitro. The novelty of this study is the biocompatibility of curcumin examined in subcutaneous connective tissue of rats using inflammatory cells as a marker of inflammation to curcumin (in vivo).

In this study, 60% of the patients possessed *P. gingivalis* in their periodontal pockets, and this is in line with the result obtained by Mayorga-Fayad and colleagues (60%) [29]. After isolation of *P. gingivalis*, its identification was confirmed by PCR because the molecular technique is more sensitive and specific than the culture method [30].

Microorganisms and inflammatory response together are the cause of many diseases, including periodontitis; hence, substances with both anti-inflammatory and antimicrobial activities are of great interest in the field of periodontology [31].

In this study, the clinically isolated strain of *P. gingivalis* was used because the laboratory strain would have been subcultured for a long time after its first isolation and might have lost its pathophysiological behaviors and therefore might not have reflected the “real-world” behavior [32]. Accordingly, the sensitivity to the antibacterial dose of curcumin found in this study is directed against a newly isolated virulent strain. Interestingly, the MIC and MBC values were equal, and this could be explained by the fact that curcumin has bactericidal rather than bacteriostatic activity against *P. gingivalis*. This supports the Bhatia et al. finding that curcumin has bactericidal activity against both *P. gingivalis and P. intermedia* [33].

The effective curcumin concentration results from our study were dissimilar and oscillating in comparison with those of previous researchers who used the *P. gingivalis* (ATCC33277) strain. In an early study in 2013, Mandroli and Bhat [20] showed that the MIC of curcumin against *P. gingivalis* (ATCC33277) was 125 *µ*g/ml. Later, Shahzad et al. [21] stated that the growth of *P. gingivalis* (ATCC33277) was inhibited by curcumin at the concentration of 7.8 *µ*g/ml, while Izui et al. [22] found that the growth of bacteria was inhibited entirely at a low concentration of curcumin of 20 *µ*g/ml in more than 40%. Recently, it was reported that *P. gingivalis* (ATCC33277) was sensitive at a higher curcumin concentration of 100 *µ*g/ml [23]. Nevertheless, all their results were higher than those reported here because of the difference in the bacterial strain used in the assessment. All previous studies measured curcumin's effect on laboratory strains, while in this study, we used clinically isolated *P. gingivalis*.

The present study confirms that curcumin can be considered as ideal for evolving into medicaments with a large extent of possible applications in periodontal therapy as a useful complementary agent because it has antibacterial properties and was proved previously to have anti-inflammatory characteristics. However, further animal and clinical studies using different curcumin concentrations are needed to improve its ability to inhibit early recolonization of periodontal pathogens in chronic periodontitis cases over a more extended period. It seems that curcumin holds a promising future in therapeutic applications in periodontology.

Many techniques have been utilized to assess the biocompatibility of dental materials. Standout and broadly used techniques are the implantations of the material into the subcutaneous connective tissue of rodents. The materials can be innovated into the tissue of animals utilizing polyethylene tubes in light of the fact that the tubes are inactive in nature which makes them an appropriate test material for contact with living tissue, and they can forestall scattering of the test materials, thus reproducing the clinical states of drugs [34, 35]. The execution of the tubes, following putting the materials in them, reproduces the genuine clinical circumstance because, even before the material is caught, they come into contact with the tissues [36]. The chafing impact of the materials can be measured by the histopathological examination of tissue reaction around the inserts [34].

**Figure 6:**
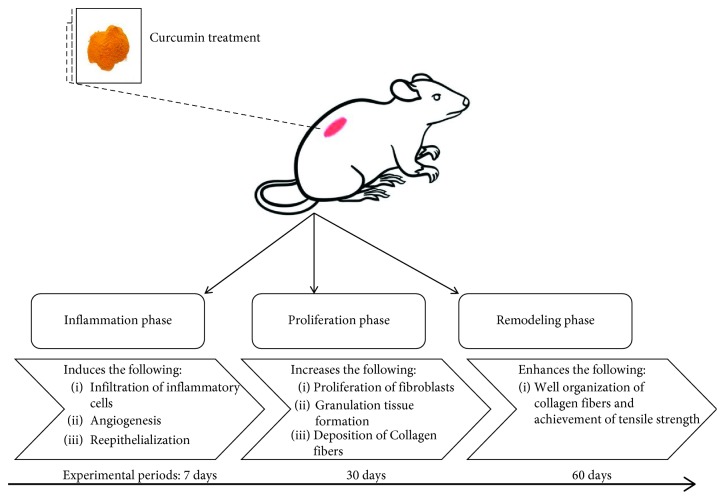
Curcumin roles in stages of wound healing.

Wound healing is a complicated method in which the skin and the tissues below it are repaired after injury. In this study, wound healing is seen in a discrete sequence of physical characteristics (stages) that constitute the posttrauma repair process [37]. Once the barrier is damaged, the injury is repaired by a controlled series of biochemical processes [37, 38]. This mechanism is classified into linear stages: blood clotting (hemostasis), inflammation, development of tissue (proliferation), and remodeling of tissue (maturation). Instead of a distinct phase, blood clotting may be regarded as part of the inflammation phase [39]. Hence, wound healing is a multistep process that comprises many stages that run from coagulation, inflammatory response, granulation tissue, a proliferation of fibroblasts, collagen formation, remodeling, and scarring [5, 40]. There is accumulating evidence that the biologic impact of curcumin is broadly considered for wound healing through its influences against inflammation, angiogenesis, and oxidation processes. The curcumin roles in relation to the healing process are shown in Figure 6.

Reactive oxygen species (ROS) are also included in wound healing as they are needed for immune system defense against microorganisms. However, the existence of ROS for a long period of time at high concentration produces oxidative stress, which can fundamentally harm human cells [5, 41]. An important factor in the wound-healing process is oxidative stress and generally restrains tissue remodeling [42]. ROS such as hydrogen peroxide (H_2_O_2_) and superoxide (O_2_^−^) can be utilized as markers for the measure of oxidative stress present in a system [43]. Like free radicals, ROS cause oxidative damage, leading to lipid peroxidation, DNA breakage, and inactivation of the enzyme, all of which repress ideal wound healing. ROS are viewed as a real reason for inflammation during wound-healing activity [44]. Lipid peroxidation is a significant procedure for wound healing. Collagen fibril viability is incremented by preventing lipid peroxidation [45].

Histological discoveries of this examination on day 7 showed that the tissue regeneration is vastly improved in skin wounds treated with tetracycline and curcumin than in control skin wounds. Despite the fact that these outcomes depend on the beneficial impacts of the treated materials on the morphology of dermal wound healing, the recently synthesized collagens were still disorganized and distributed haphazardly in all rats at this stage. The quickened wound healing in this examination by curcumin may not be because of one mechanism but rather could be due to the interchange of a few putative mechanisms. In the present study, superior healing development in rats of the curcumin-treated group might be due to the presence of polyphenolic and beta-diketone functional groups that decrease the lipid peroxidation and can improve vascularity, collagen synthesis, and advances in cross-linking of collagen because the curcumin phenolics act as strong antioxidants or free radical scavengers, and this finding is in agreement with the previous studies that assessed the histological examination of wound healing and demonstrated that the phenolic component of the curcumin can decrease harmed tissue layers and accelerate wound healing [46, 47]. Furthermore, previous studies showed that curcumin-treated local wound samples demonstrated an expansive number of inflammatory cells and fibroblasts when compared with untreated injury [48, 49] which was in accordance with the result of the current study. Migration of different cells constitutes potential sources of growth factors needed for the regulation of biological processes during wound healing.

In the present study on day 30, the results demonstrated that subcutaneous tissue facing an implanted curcumin had extensive infiltration of inflammatory cells at the initial phases of the experiment (7 and 30 days) when compared with the other two groups (polyethylene tube and tetracycline group). The curcumin had demonstrated the presence of the full-thickness epidermal layer which secured totally the wound area and prompted a broader arrangement of granulation tissue and a faster reepithelialization of the wound and more arrangement of the collagen fiber directions. These findings agree with those of previous studies [5, 48, 50]. However, it disagreed with the Mohanty et al. finding. They stated that curcumin decreases inflammation at wounded sites by the activation of the NF-kB pathway [44].

In our result, the total mean of the inflammatory cell count was significantly more in the curcumin-treated group in comparison with that in the control and tetracycline groups in both periods (*p* values = 0.002 and 0.006), and the scores were 40% severe and 80% moderate, respectively, while there was no severe score in the other two groups after 7 days and the score was 60% moderate in the control group after 30 days. This is one beneficial outcome of curcumin that was mentioned previously to cause faster healing.

The total mean numbers of the inflammatory cell count after 30 days that encompassed the opening tube were decreased in all the groups significantly except the control group which did not reach a significant level when compared to that after 7 days; also, the scores of the inflammatory reaction regressed in all groups insignificantly, and this refers to the anti-inflammatory effect of both treated groups.

The consequences of this study exhibited that the wound healing and repair in the 60 days were increasingly clear and had better remodeling in the treated groups than in the control group, especially in the rats of the curcumin-treated group, that demonstrated total reepithelialization and tissue regeneration with full maturation of the wound, well-organized epidermis with dermal papillae, well-organized thick bundles of collagen fibers, and minimum amount of inflammatory cell infiltration. In addition, a decrease in absolute cellularity and enhancing fibroblast maturation and differentiation were seen in the wound area in both treated groups which consistently with the study of Kahkeshani et al. indicated that, for a plant to be an effective wound healer, its dynamic constituents need to have anti-inflammatory, antimicrobial, and antioxidant activities. These are the significant biological activities that are focused on the development of new items for wound healing [51].

Furthermore, the presence of myofibroblasts, in curcumin-treated wound, resulted in quicker wound contraction [49]. Furthermore, curcumin results in increased fibronectin and collagen expression and increases the rate of granulation tissue formation (greater cellular content and neovascularization) and a faster reepithelialization of both diabetic wounds and those wounds impaired with hydrocortisone [52] by increasing the expression of transforming growth factor (TGF-*β*1) [53] and its receptors and nitric oxide synthase during wound healing [54].

But at the end of our experiment, the inflammatory cell counts in the curcumin group descend significantly in comparison with those in other two periods (7 and 30 days); at the same time within the same period (60 days), they remarkably decreased to a level lower than that in both comparable groups (polyethylene tube and tetracycline group), and the score of the inflammatory reaction is 100% none. This is considered an important attainment that curcumin stimulates infiltration of a large number of inflammatory cells and collagen formation at the injury site which accelerated the healing processes through rapid reepithelialization and tensile strength, and it is considered a biocompatible material since the seriousness of the connective tissue response diminishes with time [34, 55].

## 5. Conclusion

This study showed that clinically isolated *P. gingivalis* obtained from chronic periodontitis patients is highly sensitive to curcumin at a low MIC and MBC (12.5 *μ*g/ml). Curcumin is considered a biocompatible material because of reducing the severity of tissue response with time. We affirmed that local curcumin application subcutaneously increases the infiltration of inflammatory cells, cellular activation, and collagen formation and organization at the injury site which induces faster healing associated with rapid surface reepithelialization.

## Figures and Tables

**Table 1:** Frequency, morphology, and biochemical features applied for the identification of the clinically isolated *P. gingivalis* from five subgingival plaque samples.

Anaerobic *P. gingivalis*		Morphology of the pure isolated *P. gingivalis* colony			Biochemical test
Absent	Present	Pigment	Gram stain	Columbia agar	Vitek 2 system
2 (40%)	3 (60%)	Black	Gram-negative short rods	Smooth, shiny, and convex	97% probability

**Table 2:** Mean and frequency distribution of the score of inflammation severity in different groups at three experimental periods.

Period	Group	Score of inflammation severity						
		Mean ± SD	*p* value (ANOVA)	None (%)	Mild (%)	Moderate (%)	Severe (%)	*p* value
7 days	Control	1.8 ± 0.4	0.074	0	20	80	0	≤0.001
	Tetracycline	1.6 ± 0.5		0	40	60	0	
	Curcumin	2.4 ± 0.5		0	0	60	40	

30 days	Control	1.4 ± 0.9	0.016	20	20	60	0	≤0.001
	Tetracycline	0.8 ± 0.4		20	80	0	0	
	Curcumin	1.8 ± 0.4		0	20	80	0	

60 days	Control	0.4 ± 0.5	0.335	60	40	0	0	≤0.001
	Tetracycline	0.2 ± 0.4		80	20	0	0	
	Curcumin	0		100	0	0	0	

*p* value ≤0.05 was considered significant.

**Table 3:** Mean of total inflammatory cell counts in each studied group at three different periods.

Period	Control	Tetracycline	Curcumin	*p* value
7 days	64 ± 20.24	55 ± 14.44	99 ± 11.82	0.002
30 days	47.4 ± 16.8	32 ± 9.42	70.4 ± 18.2	0.006
60 days	24.2 ± 8.75	22.5 ± 7.32	15.3 ± 1.98	0.122

*p* value ≤0.05 was considered significant.

**Table 4:** Multiple comparisons depending on periods.

			Tetracycline	Curcumin	Control
Score	ANOVA		0.002	≤0.001	0.002
	7 days	30 days	0.055	0.091	0.816
	30 days	60 days	0.164	≤0.001	0.008

Cell count	ANOVA		0.001	≤0.001	0.007
	Compared groups		Post hoc tests/Tukey's HSD		
	7 days	30 days	0.015	0.010	0.267
	30 days	60 days	0.385	≤0.001	0.096

*p* value ≤0.05 was considered significant.

## Data Availability

The data used to support the findings of this study are available from the corresponding author upon request.

## Appendix

### 16S rDNA Gene Sequence

16S rDNA gene sequences are as follows:

CTGAGACGGAGGGGCGTGGGTATCAAACAGGATTAGATACCCTGGTAGTCCACGCAGTAAACGATGATTACTAGGAGTTTGCGATATACCGTCAAGCTTCCACAGCGAAAGCGTTAAGTAATCCACCTGGGGAGTACGCCGGCAACGGTGAAACTCAAAGGAATTGACGGGGGCCCGCACAAGCGGAGGAACATGTGGTTTAATTCGATGATACGCGAGGAACCTTACCCGGGATTGAAATGTAGATGACTGATGGTGAAAACCGTCTTCCCTTCGGGGCTTCTATGTAGGTGCTGCATGGTTGTCGTCAGCTCGTGCCGTGAGGTGTCGGCTTAAGTGCCATAACGAGCGCAACCCACATCGGTAGGAAAAAAAAAAGATA
